# Hypoxia impairs urothelial barrier function by inhibiting the expression of tight junction proteins in SV‐HUC‐1 cells

**DOI:** 10.1111/jcmm.18545

**Published:** 2024-07-19

**Authors:** Huijiu Luo, Hui Zhou, Yuzhu Chen, Xianwu Sun, Yihuan Li, Guangjie Li, Shouyi Long, Shiyu Wang, Guobiao Liang, Shulian Chen

**Affiliations:** ^1^ Department of Urology Affiliated Hospital of Zunyi Medical University Zunyi Guizhou China

**Keywords:** barrier function, hypoxia, tight junction, uroplakins, urothelial cells

## Abstract

Hypoxia plays an important role in the pathological process of bladder outlet obstruction. Previous research has mostly focused on the dysfunction of bladder smooth muscle cells, which are directly related to bladder contraction. This study delves into the barrier function changes of the urothelial cells under exposure to hypoxia. Results indicated that after a 5‐day culture, SV‐HUC‐1 formed a monolayer and/or bilayer of cell sheets, with tight junction formation, but no asymmetrical unit membrane was observed. qPCR and western blotting revealed the expression of TJ‐associated proteins (occludin, claudin1 and ZO‐1) was significantly decreased in the hypoxia group in a time‐dependent manner. No expression changes were observed in uroplakins. When compared to normoxic groups, immunofluorescent staining revealed a reduction in the expression of TJ‐associated proteins in the hypoxia group. Transepithelial electrical resistance (TEER) revealed a statistically significant decrease in resistance in the hypoxia group. Fluorescein isothiocyanate‐conjugated dextran assay was inversely proportional to the results of TEER. Taken together, hypoxia down‐regulates the expression of TJ‐associated proteins and breaks tight junctions, thus impairing the barrier function in human urothelial cells.

## 1 INTRODUCTION

Bladder outlet obstruction and associated lower urinary tract symptoms (BOO/LUTS) affect a large population of patients, particularly the elderly. This disorder arises from numerous conditions, such as benign prostatic hyperplasia, urethral stricture, organ prolapse and posterior urethral valves.^1^ High intravesical pressure is a characteristic of BOO, which induces decreased blood flow and oxygen deprivation in the bladder.^2^, ^3^, ^4^ Progressive tissue remodelling of the bladder includes inflammation, hypertrophy, denervation, dedifferentiation and fibrosis, which could be triggered by hypoxia and result in a decrease in compliance of the bladder.^5^, ^6^ Radford reported that hypoxia in urothelium was significantly correlated with the deterioration of bladder function in BOO patients.^7^ Many signs indicate that hypoxia plays an important role in the pathological process of the bladder.

Previous research has mostly focused on the dysfunction of bladder smooth muscle cells, which are directly related to bladder contraction.^8^ However, derived medications including α‐adrenergic receptor antagonists, muscarinic receptor inhibitors and β3‐Adrenergic receptor agonists only relieve LUTS in a small portion of patients.^9^, ^10^ Urothelial layer lining the inner part of the bladder is critical in insulating urine from smooth muscles. The barrier function of urothelial layer is to prevent the penetration of toxic solute into sub‐urothelial layer. Urothelial barrier function consists of asymmetric membrane unites (AUM) and tight junctions.

Injury of barrier function in urothelium will also result in severe LUTS, like patients of acute cystitis,^11^ where the urinary solutes (potassium, urea, toxins, etc.) are diffused into the submucosa and detrusor muscle layers, begetting hyperactivation of afferent nerve and detrusor muscle and, eventually, bladder pain, frequency and urgency of urination. Research in patients and animal models with cystitis has revealed the interruption of bladder urothelium and abnormal expression of TJ‐associated proteins.^12^, ^13^ Different from the direct inflammatory destruction of the urothelial barrier by bacteria in cystitis, whether hypoxia in BOO results in the impairment of barrier function remains to be elucidated. This study delves into the barrier function changes of the urothelial cells under exposure to hypoxia.

## 2 MATERIALS AND METHODS

### 2.1 Cell culture and exposure to hypoxia

SV‐40 immortalized human urothelial cells (SV‐HUC‐1) used in this study were purchased from ATCC. Cells were cultured in medium (high‐glucose DMEM, gibco) mixed with 10% fetal bovine serum (CY101, Cellorlab) and 1% penicillin/streptomycin (HyClone) in humidified atmosphere of 21% O_2_, 5% CO_2_ and 74% N_2_ at 37°C. Cell characteristics and the positive expression of markers CKAE1 and Upk2 were detected to confirm the urothelial phenotype. SV‐HUC‐1 cells between passages 3 and 10 were used for all experiments and were grouped when the cell density reached over 80%. In the hypoxia groups, cells were placed in a hypoxic incubator (Smartor 118, HuaYiNingChuang) with 1% O_2_, 5% CO_2_ and 94% N_2_ at 37°C for 2, 24, 48 or 72 h. An equal number of cells maintained in normoxic conditions for 2, 24, 48 or 72 h were set as controls.

### 2.2 Electron microscopy

Scanning electron microscopy (SEM) and transmission electron microscopy (TEM) were carried out to examine the TJ structure of SV‐HUC‐1 cells in hypoxia. 1.2 × 10^5^ cells were cultured in 24‐mm cover glasses through the above methods and groups. Glasses were washed with PBS at the end of the experiment, and fixed in 3.5% glutaraldehyde and 0.1 mol/L cacodylate buffer (pH 7.25) for 3 h. After post‐fixing in 1% osmium tetroxide for 2 h, cells were dehydrated by progressively higher concentrations of ethanol and embedded in epoxy resin. The cells were cut into 50‐nm thin samples and stained with toluidine blue. Cell ultrastructure was photographed with a TEM (Hitachi‐7650).

### 2.3 Quantitative real‐time PCR

Total RNA was extracted with Trizol solution (TARAKA) and transcribed into cDNA using a Reverse Transcription kit (TAKARA, RR037A). Gene amplification was performed which combined 3 μL cDNA with the primers and SYBR Fast qPCR (TAKARA, RR820) in the Biorad CFX96 real‐time system. The sequence of primers specific for target genes was as follows: ZO‐1 forward sequence (5′–3′) ATTCAATCACAATCTTCTGCCAAGT, reverse sequence (5′–3′) CCCATTGCTG TTAAATATGCCTC; claudin 1 forward sequence (5′–3′) AGATGAGGATGGCTGT CATTGGG, reverse sequence (5′–3′) CCTTGGTGTTGGGTAAGAGGTTGT; occludin forward sequence (5′–3′) GCTTCCATTAACTTCGCCTGTGG, reverse sequence (5′–3′) TTGACCTTCCTGCTCTTCCCTTT; GAPDH forward sequence (5′–3′) GGAGTCCACTGGCGTCTTCA, reverse sequence (5′–3′) GTCATGAGT‐CCTTCCACGATACC. Relative gene expression was normalized with the expression of GAPDH, and changes in different groups were expressed using the formula 2^−ΔΔCt^.

### 2.4 Western blot (WB) analysis

Target cells collected from each group were lysed with RIPA buffer (Solarbio), and the protein concentration was detected using BCA Assay Kit (Solarbio). Equal number of proteins were isolated by 10% SDS‐PAGE and transferred on PVDF membranes. After blocked by 5% BSA for 2 h, the membranes were incubated in primary antibodies overnight at 4°C. Then, the membranes were washed with TBS and incubated in secondary antibodies for 2 h at room temperature. The antibodies were as follows: rabbit anti‐ZO‐1 polyclonal antibody (1:500, Abcam); rabbit anti‐claudin1 polyclonal antibody (1:500, Abcam); rabbit anti‐occludin polyclonal antibody (1: 500, Abcam); vimentin (1:1000, Abcam); tubulin (1:2000, Abcam); goat anti‐rabbit IgG H&L (1:2000, Abcam). Protein bands were visualized by using Omni‐ECL kit (Epizyme). Image J analysis software was applied to analyse the grey values of relevant protein bands.

### 2.5 Immunofluorescent (IF) staining

The cell samples were fixed with 4% paraformaldehyde for 15 min, and permeabilised with 0.3% Triton X‐100 for 30 min. Blocked by 5% BSA for 1 h, cells were incubated using primary antibodies (1:100, Abcam) at 4°C overnight, and were then incubated in Alexa Fluor 488‐conjugated goat anti‐rabbit IgG anti‐body (1:500, Huabio) for 1 h in the dark. Cell nuclear were stained with 4′,6‐diamidino‐2‐phenylindole (DAPI) for 15 min in the dark. Images of the stained cells were observed under inverted fluorescence microscope (IX73, Olympus).

### 2.6 Transepithelial electrical resistance (TEER) measurement

SV‐HUC‐1 cells were seeded at 2 × 10^4^/insert in 12‐mm diameter Transwell inserts. After 5‐day culture, the cells were subjected to hypoxic or normoxic condition according to the set time points. Then, the TEER was measured by an Epithelial Voltohmmeter (EVOM, World Precision Instruments, Inc., Sarasota, FL).

### 2.7 Permeability assay

A fluorescein isothiocyanate (FITC)‐labelled dextran (MW3000) was used. SV‐HUC‐1 cells were seeded at 2 × 10^4^/insert in 12‐mm diameter Transwell inserts. After 5‐day culture, the TEER reached a plateau. Then, the cells were subjected to hypoxic or normoxic conditions according to the set time points. The FITC‐dextran (1 mg/mL) was added to the apical side of the monolayer cells and 1 mL tracer‐free medium was added to the basal chamber. The culture was incubated for 3 h. Then, 300 μL of the medium was collected from the basal chamber and analysed by fluorimetry.

### 2.8 Statistical analysis

All experiments were independently repeated more than three times. Graphpad Prism 6.0 was used to perform statistical analysis. Two‐way anova was applied to evaluate differences between multiple groups and the paired *t*‐test was applied for pairwise comparison between the hypoxic group and the normoxic group in the same period. All values were expressed as mean ± standard error and *p* value < 0.05 was considered statistically significant.

## 3 RESULTS

### 3.1 SV‐HUC cells developed tight junctions after 5‐day culture

**FIGURE 1:**
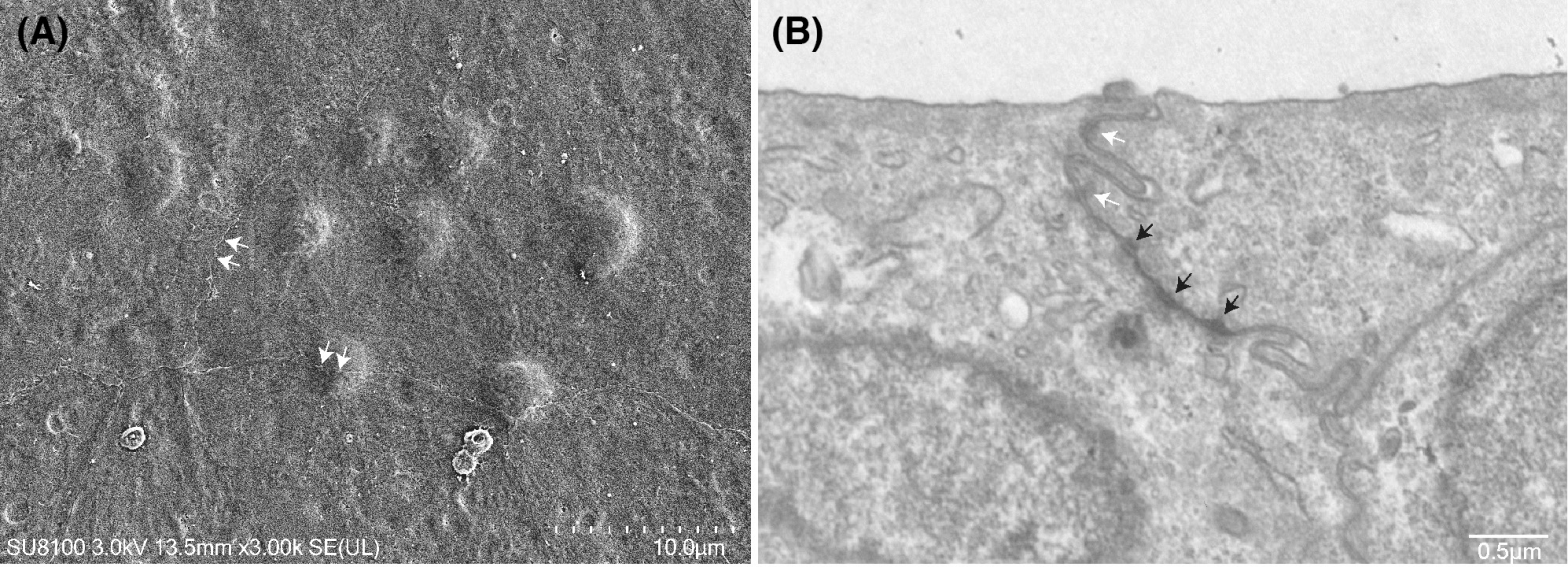
Electron microscopy images of SV‐40 immortalized human urothelial cells (SV‐HUC‐1 cells). (A) Scanning electron microscopy (SEM) image of the apical surface of SV‐HUC‐1 cells after 5‐day culture. The SV‐HUC‐1 cells were tightly apposed; (B) transmission electron microscopy (TEM) micrograph revealed the formation of tight junctions (white arrows) and adherens junctions (black arrows).

SV‐HUC‐1 at passages 3–7 showed typical flattened cells with an irregular outline in the micrographs. SEM micrograph indicated the surface of tightly apposed SV‐HUC‐1 cells, and white arrows suggested intracellular boundaries (Figure 1A). TEM micrograph showed the formation of tight junctions and adherens junctions (Figure 1B). Many clear cytoplasmic vesicles clustered together close to the apical side in the cells.

### 3.2 Hypoxia down‐regulated the mRNA expression of TJ‐ proteins in SV‐HUC‐1 cells

**FIGURE 2:**
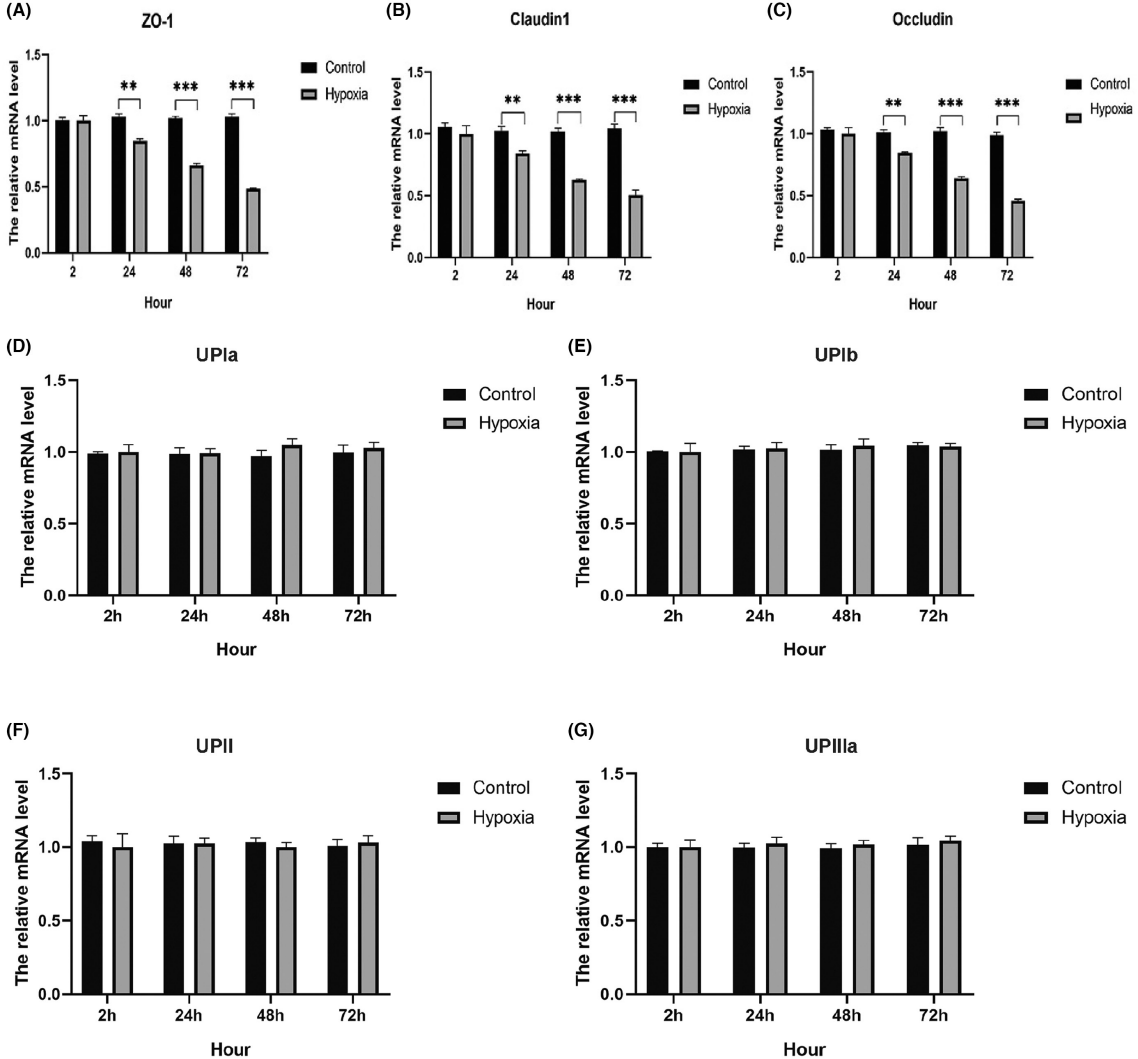
Effect of hypoxia on the urothelial barrier‐related gene expression. (A–C) Gene expression of ZO‐1, claudin1 and occludin was noticeably decreased under exposure to hypoxia; (D–G) no significant changes were found on the gene expression of UPIa, UPIb, UPII and UPIIIa (*p < 0.05, **p < 0.01, **p < 0.001).

To investigate the influence of hypoxia on the barrier‐formation proteins, qPCR was used to measure the mRNA expression of AUM proteins (UPIa, UPIb, UPII and UPIIIa) and TJ‐associated proteins (ZO‐1, claudin1 and occludin). Temporary oxygen deprivation (within 2 h) did not change the expression of TJ‐associated proteins. However, when hypoxic exposure extended to 24 h, ZO‐1, claudin and occludin mRNA decreased significantly with time (Figure 2A–C). On the contrary, hypoxic treatment did not alter the mRNA level of AUM proteins (Figure 2D–G).

### 3.3 Hypoxic exposure damaged TJ structure in SV‐HUC‐1 cells

**FIGURE 3:**
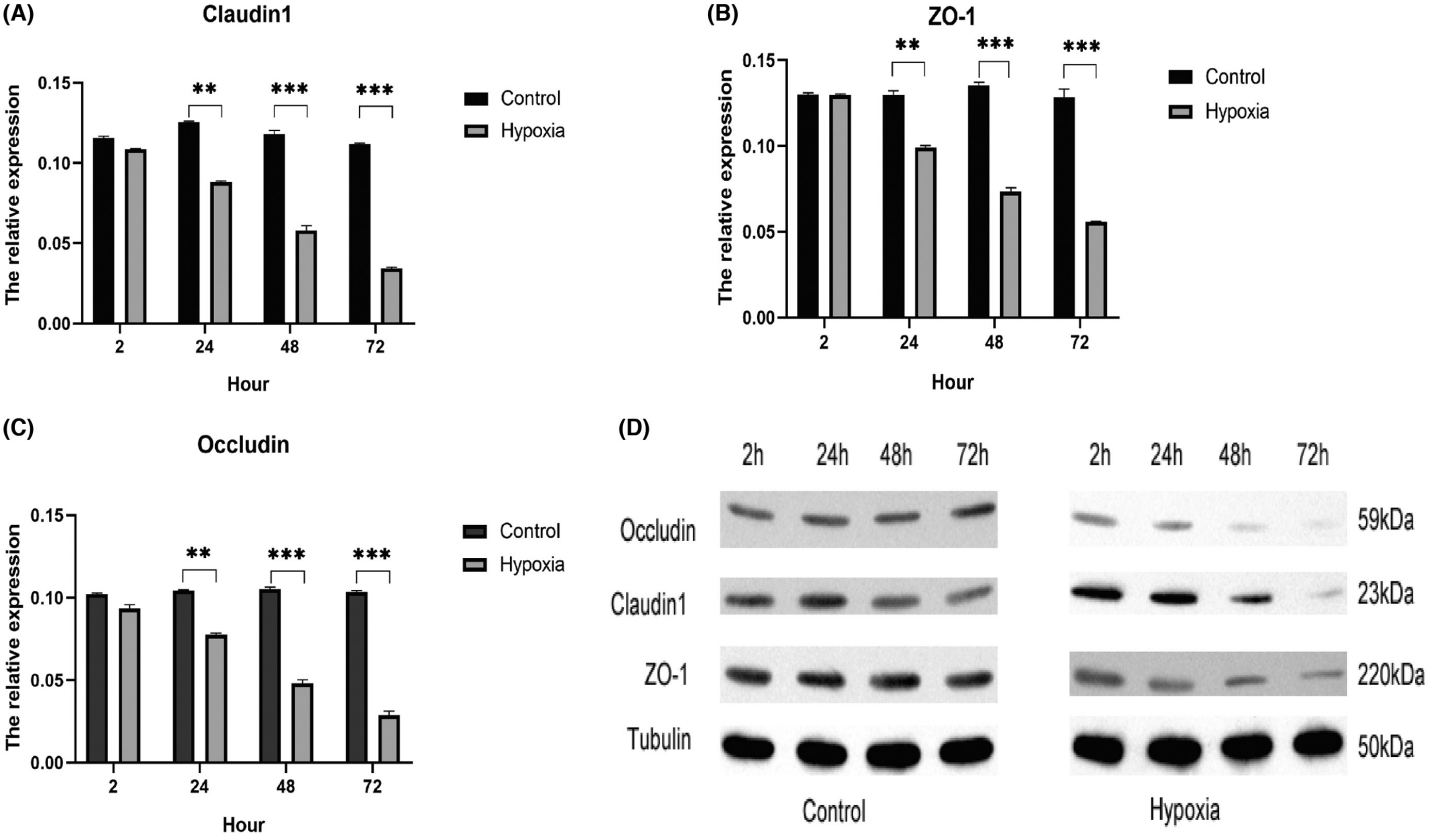
Effect of hypoxia on the protein expression of TJ‐related proteins. (A–C) Protein expression of claudin1, ZO‐1 and occludin was time‐dependently reduced in hypoxia group; (D) bands of western blot (*p < 0.05, **p < 0.01, **p < 0.001).

**FIGURE 4:**
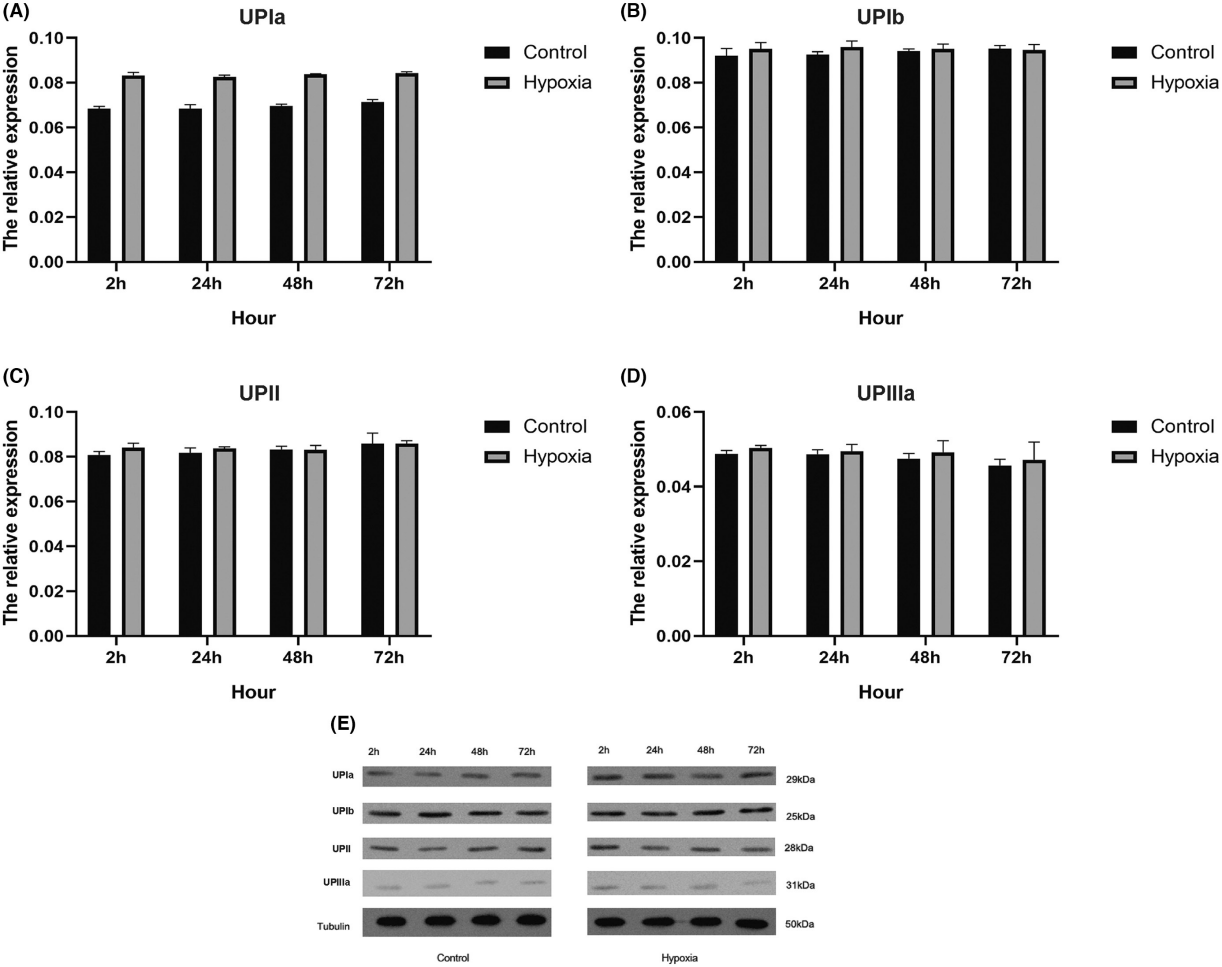
Effect of hypoxia on the protein expression of asymmetric membrane unites (AUM)‐related proteins. (A–D) No significant changes were detected on the densitometric quantification of UPIa, UPIb, UPII and UPIIIa; (E) bands of western blot.

**FIGURE 5:**
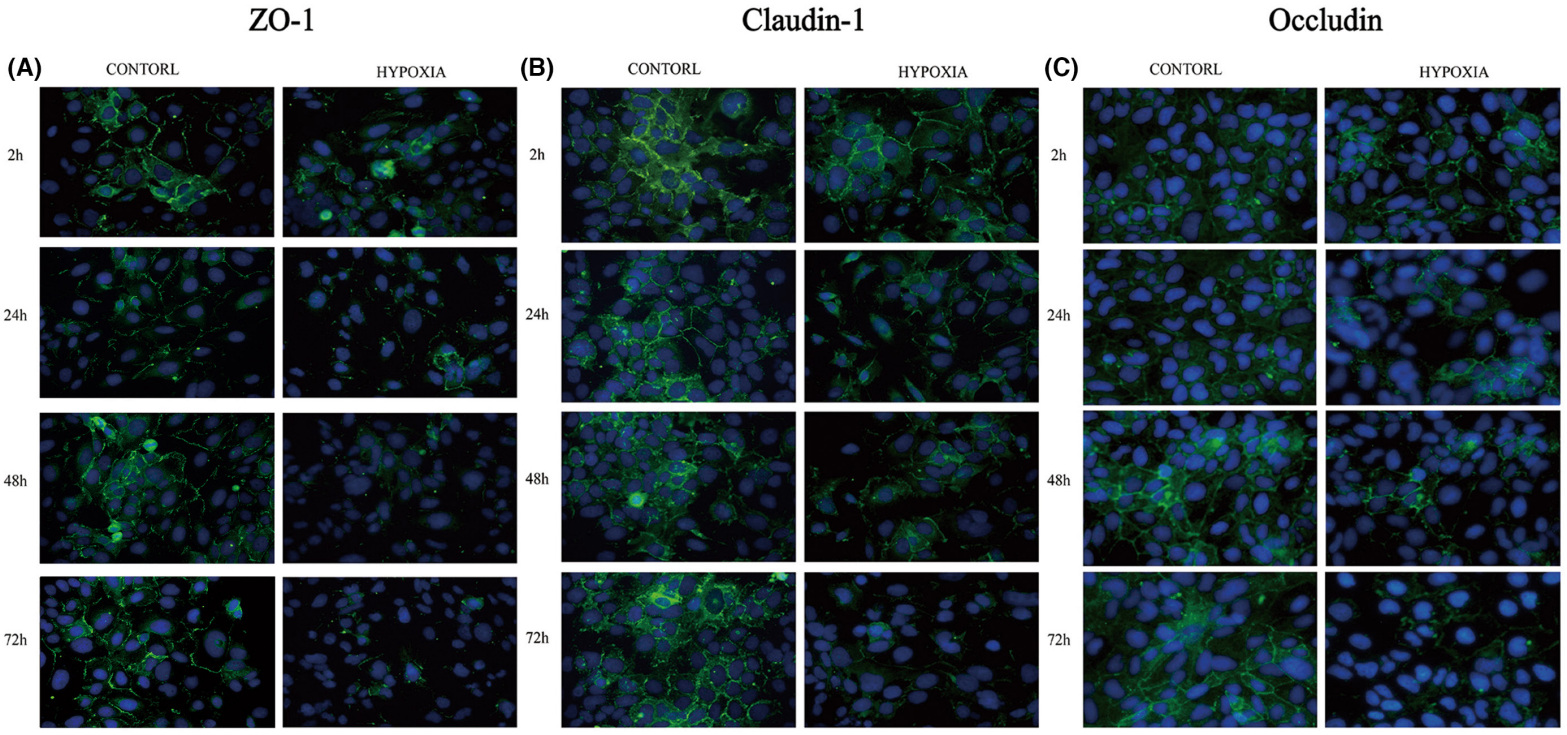
Immunofluorescent staining of ZO‐1, claudin‐1 and occludin proteins under exposure to hypoxia. (A–C) Hypoxia sharply down‐regulated the protein expression of ZO‐1, claudin‐1 and occludin.

WB assay further detected the protein level of AUM proteins and TJ‐associated proteins. When compared with the normoxic group, no significant change was found under 2‐h hypoxic treatment. However, 24‐h and more hypoxic exposure significantly lowered the protein expression of TJ‐associated proteins. Herein, occludin had the most pronounced decrease (Figure 3). Same as mRNA results, protein expression of AUM—UPIa, UPIb, UPII and UPIIIa—did not change much (Figure 4). Further IF staining was employed to observe TJ‐associated proteins. IF staining demonstrated an increase in claudin‐1 and occludin protein expression with time. However, in the hypoxia group, attenuated expression of ZO‐1, claudin‐1 and occludin protein along the cell–cell junctions was found (Figure 5).

### 3.4 Hypoxia reduced the TEER in SV‐HUC‐1 cells

**FIGURE 6:**
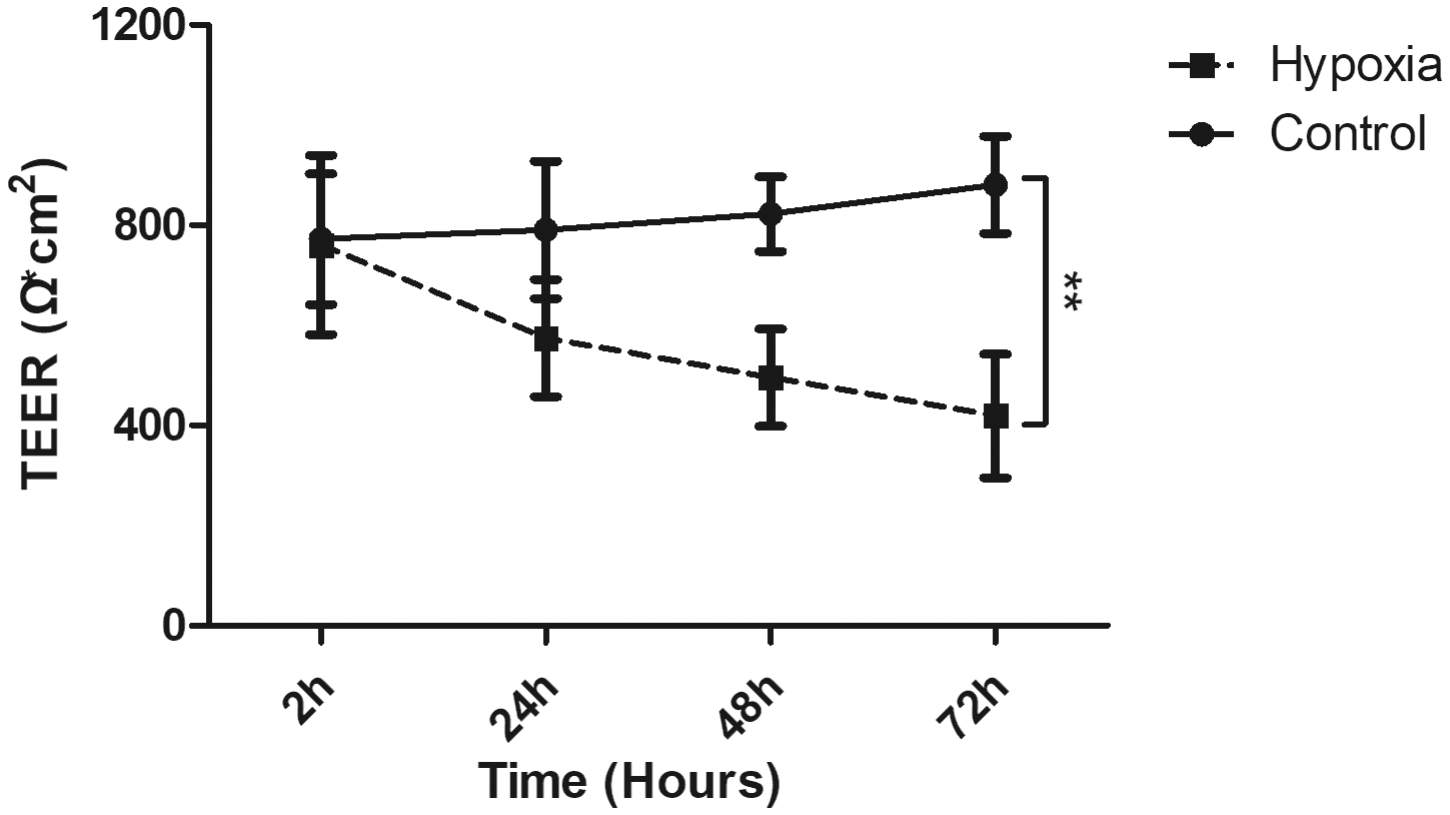
Influence of hypoxia on the transepithelial electrical resistance (TEER) of SV‐HUC‐1 cells. The graph showed time‐dependently decreased TEER in the hypoxia group (**p < 0.01).

After 5‐day culture, SV‐HUC‐1 cells formed a monolayer of cell sheets and the TEER reached a plateau of around 800 Ω/cm^2^. TEER in the normoxic group increased slightly. However, the TEER decreased gradually to approximately half of the matching group (about 400 Ω/cm^2^) on the third day of hypoxic treatment. The TEER of SV‐HUC‐1 cells was significantly diminished after 72‐h hypoxic treatment (Figure 6).

### 3.5 Hypoxia time‐dependently increased the permeability of SV‐HUC‐1 cell culture

**FIGURE 7:**
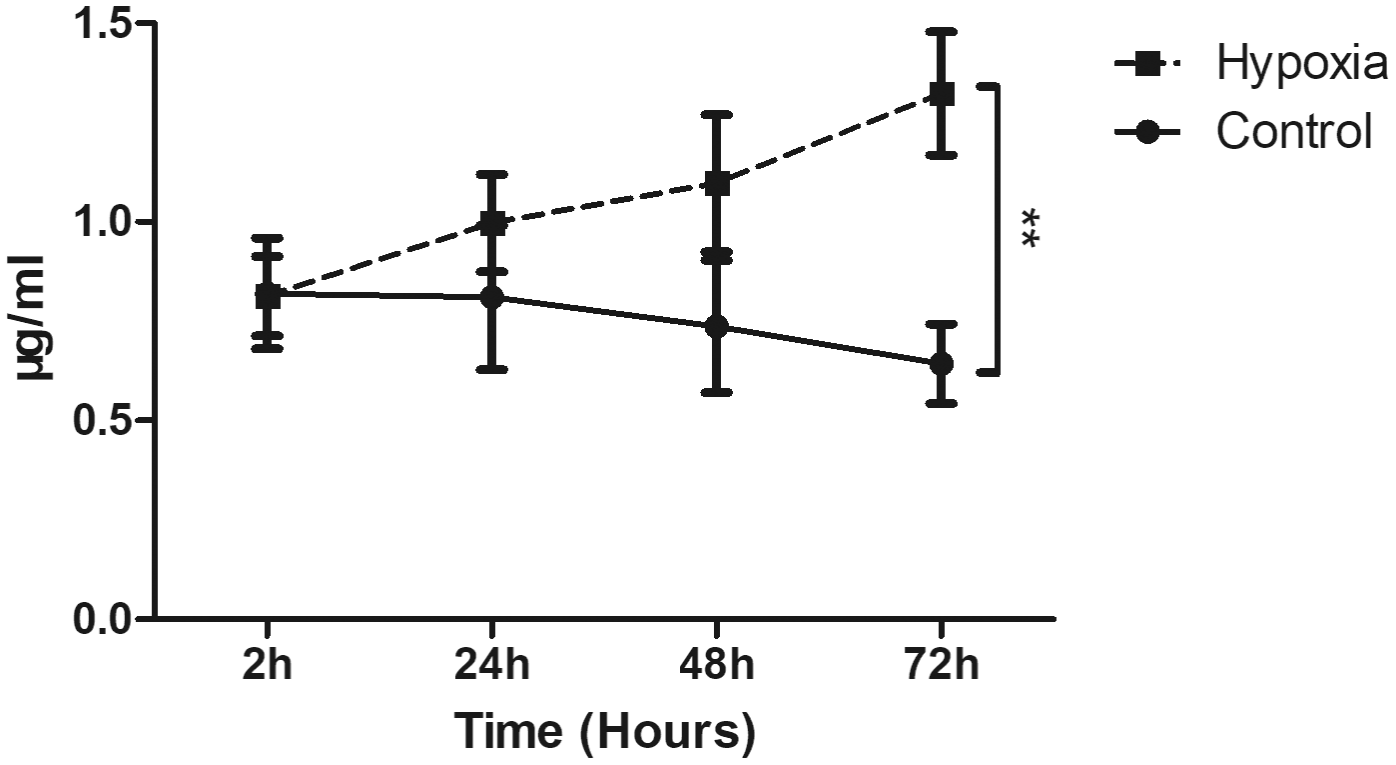
Permeability assay of SV‐40 immortalized human urothelial cells (SV‐HUC‐1). A vertical axis indicates fluorescein isothiocyanate (FITC)‐labelled dextran concentration (μg/mL). A horizontal axis shows the time after 5‐day incubation of SV‐HUC‐1. The graph shows hypoxia significantly increased the permeability of SV‐HUC‐1 (**p < 0.01).

After 5 days of incubation, the permeability of SV‐HUC‐1 cells was sustained at a low level around 0.82 μg/mL in the lower chamber of the insert. The permeability assay was used to further assess the barrier function at specific time points after hypoxic exposure. There is an increase in the concentration of FITC‐labelled dextran in the basal chamber over time after hypoxic treatment. However, a significant change was observed over 72‐h hypoxic exposure. Prolonged hypoxic exposure elevated the permeability of SV‐HUC‐1 cells (Figure 7).

## 4 DISCUSSION

Urothelial barrier is the most impermeable and the tightest barrier in a human body with a transepithelial resistance as high as 75,000 Ω/cm^2^, which is of great importance for bladder function and metabolic balance.^14^ AUM and tight junctions contribute to the formation of urothelial barrier. Disruption of either of the structures leads to the leakage of toxic solute into the submucosal layer, which would trigger LUTS. Our data found hypoxia reduced the expression of TJ‐associated proteins, attenuated TJs, decreased TEER and increased permeability of the urothelium. Although the hypoxic treatment did not change the protein expression of urothelial plaques, a decrease in TJ would increase intercellular leakage, directly irritating submucosal nerves and detrusor muscles. These changes will finally beget LUTS. Our results indicate TJ attenuation and permeability increase may be important pathophysiological alterations in bladder hypoxia disorders, such as BOO and elderly‐related OAB.

TJs are formed between adjacent umbrella cells adjacent to the apical side. ZO‐1, occludin and claudin are the main components of this structure. Claudins mediate the pore pathway of small ion and solute movement. Occludin and ZO‐1 mediate the leakage pathway of large solute movement.^11^ When the TJ‐associated proteins are damaged by some pathological factors, the barrier will be undermined with the enhanced pore and leakage pathways of the urothelium. In this study, three functional proteins were down‐regulated under exposure to hypoxia when compared with the controls. Our findings are consistent with those in the endothelial cells and trophoblast cells suggesting that hypoxia could impair barrier function and increase the permeability by downregulation of TJ proteins, such as ZO‐1 and occludin.^15^, ^16^, ^17^ This result was further confirmed by a SD rat model of middle cerebral artery occlusion.^18^ However, the research of Stephane Chabaud indicated hypoxia improved urothelial cell expansion and differentiation, thereby decreasing the permeability of bladder mucosa substitutes. The use of different cell types and oxygen concentrations could be the cause of this divergence. Besides, hypoxia may be beneficial to basal cells included in Chabaud's study, but not for differentiated cells in our research.^19^

This study also observed the changes of AUM and uroplakins in SV‐HUC‐1 cells under hypoxia. As a highly specialized structure in the urothelium, AUM was not found by TEM in this experiment. This may be attributed to the incorrect assembly of uroplakins, the chief components of AUM particles. In the urothelium, the formation of AUM particles depends on the precise assembly of uroplakins including Upk1A, Upk1B, Upk2 and Upk3A, in which glycosylation plays an important role. Studies have shown that AUM cannot be formed in cultured bovine urothelial cells due to the differentiation‐dependent glycosylation of Upk2.^20^ Four uroplakins were detected by PCR and WB. Still, we did not observe a significant expression difference between the normoxic group and the hypoxic group. Some animal experiments found that nicotine‐induced hypoxia decreased the expression of UPIII,^21^ but the influence of nicotine could not be ruled out in their results. Our research was the first data that demonstrated the effect of hypoxia on the expression of uroplakins in SV‐HUC‐1 cells.

Urothelial barrier dysfunction is believed to be a reason for storage LUTS, originating from increased permeability for solutes such as urea or potassium.^22^, ^23^ This scenario was found in BOO Wistar rats, with a decreased density of tight junctions.^24^ Moreover, intracellular tight junctions with the function of a urine–blood barrier are essential to innate immunity. Disruption of this ultrastructure predisposes to urinary tract infections and chronic bladder inflammation.^25^, ^26^ Our data demonstrated that hypoxia is an important pathophysiological alteration that affects tight junctions and bladder permeability, which may provide a novel direction for early protection of bladder function in BOO.

There are also some limitations in this study. One of them is that 2‐dimensional culture disturbed interactions among urothelium, suburothelium and extracellular environments, which may undermine the results.^27^ Bladder organoids may provide a better model for researching bladder disorders.^28^ Another limitation is that we did not investigate the underlying pathways of TJ changes caused by hypoxia. Hopefully, further research will address these issues, which may promote our understanding of urothelial barrier dysfunction in BOO.

## 5 CONCLUSIONS

Hypoxia downregulates the expression of TJ‐associated proteins and impairs tight junction, thus increasing permeability in human urothelial cells. We provide new evidence for urothelial barrier dysfunction in patients with BOO/LUTS which may become a potential target for alleviating LUTS.

## AUTHOR CONTRIBUTIONS

**Huijiu Luo:** Conceptualization (equal); data curation (equal); formal analysis (equal); methodology (equal); resources (equal); writing – original draft (equal). **Hui Zhou:** Conceptualization (equal); data curation (equal); methodology (equal); project administration (equal); writing – original draft (equal). **Yuzhu Chen:** Project administration (equal). **Xianwu Sun:** Project administration (equal). **Yihuan Li:** Project administration (equal). **Guangjie Li:** Project administration (equal). **Shouyi Long:** Methodology (equal). **Shiyu Wang:** Formal analysis (equal). **Guobiao Liang:** Formal analysis (equal); project administration (equal); supervision (equal); writing – review and editing (equal). **Shulian Chen:** Formal analysis (equal); project administration (equal); supervision (equal); writing – review and editing (equal).

## FUNDING INFORMATION

This study was supported by the National Natural Science Foundation of China (grant no. 81960148), the Guizhou Science and Technology Department (grant no. ZK2021380) and the Doctoral Foundation of the Affiliated Hospital of Zunyi Medical University (grant no. 201801).

## CONFLICT OF INTEREST STATEMENT

The authors declare no conflict of interest.

## Data Availability

The data that support the findings of this study are available from the corresponding author upon reasonable request.

## References

[1] Spigt MG , van Schayck CP , van Kerrebroeck PE , van Mastrigt R , Knottnerus JA . Pathophysiological aspects of bladder dysfunction: a new hypothesis for the prevention of ‘prostatic’ symptoms. Med Hypotheses. 2004;62(3):448‐452. doi:10.1016/j.mehy.2003.10.004 14975521

[2] Kershen RT , Azadzoi KM , Siroky MB . Blood flow, pressure and compliance in the male human bladder. J Urol. 2002;168(1):121‐125. 12050504

[3] Farag FF , Meletiadis J , Saleem MD , Feitz WF , Heesakkers JP . Near‐infrared spectroscopy of the urinary bladder during voiding in men with lower urinary tract symptoms: a preliminary study. Biomed Res Int. 2013;2013:452857. doi:10.1155/2013/452857 23936801 PMC3725978

[4] Koritsiadis G , Stravodimos K , Koutalellis G , et al. Immunohistochemical estimation of hypoxia in human obstructed bladder and correlation with clinical variables. BJU Int. 2008;102(3):328‐332. doi:10.1111/j.1464-410X.2008.07593.x 18384635

[5] Wiafe B , Adesida A , Churchill T , Adewuyi EE , Li Z , Metcalfe P . Hypoxia‐increased expression of genes involved in inflammation, dedifferentiation, pro‐fibrosis, and extracellular matrix remodeling of human bladder smooth muscle cells. In Vitro Cell Dev Biol Anim. 2017;53(1):58‐66. doi:10.1007/s11626-016-0085-2 27632054

[6] Fusco F , Creta M , de Nunzio C , et al. Progressive bladder remodeling due to bladder outlet obstruction: a systematic review of morphological and molecular evidences in humans. BMC Urol. 2018;18(1):15. doi:10.1186/s12894-018-0329-4 29519236 PMC5844070

[7] Radford A , Hinley J , Pilborough A , Southgate J , Subramaniam R . Hypoxic changes to the urothelium as a bystander of end‐stage bladder disease. J Pediatr Urol. 2019;15(2):158.e1‐158.e10. doi:10.1016/j.jpurol.2019.01.012 30862459

[8] Bellucci CHS , Ribeiro WO , Hemerly TS , et al. Increased detrusor collagen is associated with detrusor overactivity and decreased bladder compliance in men with benign prostatic obstruction. Prostate Int. 2017;5(2):70‐74. doi:10.1016/j.prnil.2017.01.008 28593170 PMC5448720

[9] Majima T , Matsukawa Y , Funahashi Y , Kato M , Yamamoto T , Gotoh M . The effect of mirabegron on bladder blood flow in a rat model of bladder outlet obstruction. World J Urol. 2020;38(8):2021‐2027. doi:10.1007/s00345-019-02939-9 31664511

[10] Novara G , Galfano A , Ficarra V , Artibani W . Anticholinergic drugs in patients with bladder outlet obstruction and lower urinary tract symptoms: a systematic review. Eur Urol. 2006;50(4):675‐683. doi:10.1016/j.eururo.2006.07.017 16930813

[11] Monaco A , Ovryn B , Axis J , Amsler K . The epithelial cell leak pathway. Int J Mol Sci. 2021;22(14):7677. doi:10.3390/ijms22147677 34299297 PMC8305272

[12] Hauser PJ , Dozmorov MG , Bane BL , Slobodov G , Culkin DJ , Hurst RE . Abnormal expression of differentiation related proteins and proteoglycan core proteins in the urothelium of patients with interstitial cystitis. J Urol. 2008;179(2):764‐769. doi:10.1016/j.juro.2007.09.022 18082196 PMC2652890

[13] Chen YH , Chen CJ , Wang SJ , et al. Downregulation of tight junction protein zonula occludens‐2 and urothelium damage in a cyclophosphamide‐induced mouse model of cystitis. Taiwan J Obstet Gynecol. 2018;57(3):399‐406. doi:10.1016/j.tjog.2018.04.013 29880173

[14] Kreft ME , Hudoklin S , Jezernik K , Romih R . Formation and maintenance of blood‐urine barrier in urothelium. Protoplasma. 2010;246(1–4):3‐14. doi:10.1007/s00709-010-0112-1 20521071

[15] Yan SF , Ogawa S , Stern DM , Pinsky DJ . Hypoxia‐induced modulation of endothelial cell properties: regulation of barrier function and expression of interleukin‐6. Kidney Int. 1997;51(2):419‐425. doi:10.1038/ki.1997.56 9027716

[16] Ma X , Zhang H , Pan Q , et al. Hypoxia/Aglycemia‐induced endothelial barrier dysfunction and tight junction protein downregulation can be ameliorated by citicoline. PLoS One. 2013;8(12):e82604. doi:10.1371/journal.pone.0082604 24358213 PMC3865100

[17] Zhang Y , Zhao HJ , Xia XR , et al. Hypoxia‐induced and HIF1α‐VEGF‐mediated tight junction dysfunction in choriocarcinoma cells: implications for preeclampsia. Clin Chim Acta. 2019;489:203‐211. doi:10.1016/j.cca.2017.12.010 29223764

[18] Chen XY , Wan SF , Yao NN , et al. Inhibition of the immunoproteasome LMP2 ameliorates ischemia/hypoxia‐induced blood‐brain barrier injury through the Wnt/β‐catenin signalling pathway. Mil Med Res. 2021;8(1):62. doi:10.1186/s40779-021-00356-x 34857032 PMC8641178

[19] Chabaud S , Saba I , Baratange C , et al. Urothelial cell expansion and differentiation are improved by exposure to hypoxia. J Tissue Eng Regen Med. 2017;11(11):3090‐3099. doi:10.1002/term.2212 28156053

[20] Hu CC , Liang FX , Zhou G , et al. Assembly of urothelial plaques: tetraspanin function in membrane protein trafficking. Mol Biol Cell. 2005;16(9):3937‐3950. doi:10.1091/mbc.e05-02-0136 15958488 PMC1196309

[21] Nagai T , Imamura T , Ogawa T , et al. Nicotine‐induced hypoxia in rat urothelium deteriorates bladder storage functions. Neurourol Urodyn. 2019;38(6):1560‐1570. doi:10.1002/nau.24050 31194269

[22] Parsons CL . The role of a leaky epithelium and potassium in the generation of bladder symptoms in interstitial cystitis/overactive bladder, urethral syndrome, prostatitis and gynaecological chronic pelvic pain. BJU Int. 2011;107(3):370‐375. doi:10.1111/j.1464-410X.2010.09843.x 21176078

[23] Niemczyk G , Czarzasta K , Radziszewski P , Włodarski P , Cudnoch‐Jędrzejewska A . Pathophysiological effect of bladder outlet obstruction on the urothelium. Ultrastruct Pathol. 2018;42(3):317‐322. doi:10.1080/01913123.2018.1462874 29671672

[24] Celik‐Ozenci C , Ustunel I , Erdogru T , et al. Ultrastructural and immunohistochemical analysis of rat uroepithelial cell junctions after partial bladder outlet obstruction and selective COX‐2 inhibitor treatment. Acta Histochem. 2006;107(6):443‐451. doi:10.1016/j.acthis.2005.09.004 16253314

[25] Spencer JD , Schwaderer AL , Becknell B , Watson J , Hains DS . The innate immune response during urinary tract infection and pyelonephritis. Pediatr Nephrol. 2014;29(7):1139‐1149. doi:10.1007/s00467-013-2513-9 23732397 PMC3800267

[26] Liu HT , Jiang YH , Kuo HC . Alteration of urothelial inflammation, apoptosis, and junction protein in patients with various bladder conditions and storage bladder symptoms suggest common pathway involved in underlying pathophysiology. Low Urin Tract Symptoms. 2015;7(2):102‐107. doi:10.1111/luts.12062 26663690

[27] Kapałczyńska M , Kolenda T , Przybyła W , et al. 2D and 3D cell cultures—a comparison of different types of cancer cell cultures. Arch Med Sci. 2018;14(4):910‐919. doi:10.5114/aoms.2016.63743 30002710 PMC6040128

[28] Kim E , Choi S , Kang B , et al. Creation of bladder assembloids mimicking tissue regeneration and cancer. Nature. 2020;588(7839):664‐669. doi:10.1038/s41586-020-3034-x 33328632

